# Improved accuracy of multiple ncRNA alignment by incorporating structural information into a MAFFT-based framework

**DOI:** 10.1186/1471-2105-9-212

**Published:** 2008-04-25

**Authors:** Kazutaka Katoh, Hiroyuki Toh

**Affiliations:** 1 Digital Medicine Initiative, Kyushu University, Fukuoka, 812-8582, Japan; 2 Medical Institute of Bioregulation, Kyushu University, Fukuoka, 812-8582, Japan

## Abstract

**Background:**

Structural alignment of RNAs is becoming important, since the discovery of functional non-coding RNAs (ncRNAs). Recent studies, mainly based on various approximations of the Sankoff algorithm, have resulted in considerable improvement in the accuracy of pairwise structural alignment. In contrast, for the cases with more than two sequences, the practical merit of structural alignment remains unclear as compared to traditional sequence-based methods, although the importance of multiple structural alignment is widely recognized.

**Results:**

We took a different approach from a straightforward extension of the Sankoff algorithm to the multiple alignments from the viewpoints of accuracy and time complexity. As a new option of the MAFFT alignment program, we developed a multiple RNA alignment framework, X-INS-i, which builds a multiple alignment with an iterative method incorporating structural information through two components: (1) pairwise structural alignments by an external pairwise alignment method such as SCARNA or LaRA and (2) a new objective function, Four-way Consistency, derived from the base-pairing probability of every sub-aligned group at every multiple alignment stage.

**Conclusion:**

The BRAliBASE benchmark showed that X-INS-i outperforms other methods currently available in the sum-of-pairs score (SPS) criterion. As a basis for predicting common secondary structure, the accuracy of the present method is comparable to or rather higher than those of the current leading methods such as RNA Sampler. The X-INS-i framework can be used for building a multiple RNA alignment from any combination of algorithms for pairwise RNA alignment and base-pairing probability. The source code is available at the webpage found in the Availability and requirements section.

## Background

Multiple alignment is an important step in various phases of comparative studies of RNAs, such as the detection of common secondary structures from a set of homologous sequences and the preparation of an alignment as a query for database search tools including Infernal [1]. Since the discovery of functional non-coding RNAs (ncRNAs), the necessity for the incorporation of secondary structural information into a multiple RNA alignment has been recognized, and many efforts are being made toward this goal [2-14]. Secondary structure prediction and multiple RNA alignment are closely related to each other. According to Gardner and Giegerich [15], there are three possible plans to infer common secondary structures from a set of unaligned RNA sequences, align-then-fold (plan A), simultaneous (plan B) and fold-then-align (plan C). Plan B generally has a higher computational cost than plans A and C.

The Sankoff algorithm, which simultaneously performs sequence alignment and secondary structure prediction, is available for plan B. This algorithm is not applicable to real analyses with more than two sequences due to its time complexity, *O*(*L*^3*N*^), where *L *is the sequence length and *N *is the number of sequences. Even if a virtually optimum pairwise structural alignment were successfully obtained by using variants of the Sankoff algorithm or by other algorithms, handling multiple sequences would remain a nontrivial task. The situation would be similar to that of a pure sequence alignment problem, for which the optimum solution is exactly calculated by using a two-dimensional dynamic programming (DP) algorithm [16,17] in cases with two sequences. With an increased number of sequences, it becomes difficult to obtain the exactly optimum solution. For predicting common secondary structure in multiple unaligned sequences, some sort of heuristics will inevitably be required, since an exact application of plan B is impossible.

Judging from previous studies on multiple sequence alignment, obtaining the optimum solution in plan B is not the most important issue, from a biological viewpoint. It is known that the optimum solution of a multiple sequence alignment problem is not always the correct one [18,19]. This suggests that we should pay attention to a biologically relevant objective function as well as to algorithmic techniques for obtaining the optimum solution. This is one of the reasons why various multiple sequence alignment schemes have been intensively studied to date, and why there are no definitive ones yet. Moreover, the accuracy of multiple alignment is improved by using information of homologs, probably because homologs make family-specific information available and enrich the profiles used in the multiple alignment processes [20-26]. This point is possibly related to the strange observation, reported in Bauer *et al*. [9], that a purely sequence-based method, MAFFT-G-INS-i [22], performed better with a growing number of input sequences; in cases with 10 or 15 sequences, it even outperformed structural alignment methods, except for LaRA.

Since the application of plan B to multiple RNAs has both computational and biological difficulties, as explained above, various structural alignment techniques have been developed, partly or wholly incorporating plans A and C. For example, LaRA [9] constructs a set of pairwise structural alignments by a novel graph-based approach and subjects them, as a primary library, to the T-Coffee sequence alignment program [27] to construct a multiple structural alignment. MXSCARNA [14] is a multiple alignment extension of a rapid pairwise structural alignment algorithm, SCARNA [28], which is based on fixed-length stem fragments as the representation of secondary structure. These two methods employ plan B with novel techniques at the pairwise alignment stage, while constructing a multiple alignment with consistency-based techniques [27,29] originally developed for multiple sequence alignment. The common secondary structures are predicted from the resulting multiple alignment, as in plan A. In contrast, the combination of RNAcast and RNAforester [3] can be classified into plan C. RNA sampler [11] takes a different approach, in which the common structures between each pair of sequences are probabilistically sampled and iteratively updated. At the cost of CPU time, RNA Sampler achieved a remarkable improvement in accuracy.

Based on these and other previous studies, we sought to develop a multiple global alignment method that efficiently utilizes structural information at the multiple alignment stage. We focused on the multiple global alignment of ncRNAs, rather than similarity searches and motif finding by local alignment. As a starting point, we selected MAFFT-G-INS-i, since it was reportedly the most accurate among the sequence-based alignment methods for globally alignable ncRNAs in the BRAliBASE benchmark test [30]. As no structural information is considered in G-INS-i, there could be room for further improvement in the accuracy. Hence, we developed a new objective function, Four-way Consistency, that is calculated from the base-pairing probability of every aligned group at each multiple alignment step. It was implemented in a new option of MAFFT, X-INS-i, which loads pairwise structural alignments computed by an external pairwise alignment method, such as SCARNA [28] and LaRA [9], and combines them into a single multiple alignment through a progressive method and a subsequent iterative refinement method, based on the new objective function. To facilitate rapid computation, the objective function was designed to have an affinity to a group-to-group alignment algorithm based on DP.

X-INS-i can be classified as an intermediate plan between B and C: it adopts plan B at the pairwise alignment stage, while the base-pairing probability calculated from individual sequences is used, as in plan C, at the multiple alignment stage. We also introduced a simplified variant, Q-INS-i, which can be classified into plan C. Q-INS-i uses a purely sequence-based pairwise alignment algorithm, instead of a pairwise structural alignment algorithm, together with the Four-way Consistency objective function, calculated from the base-pairing probability of each sequence. As both X-INS-i and Q-INS-i simply output a multiple sequence alignment, they require, like plan A, an external program to predict a common secondary structure, such as RNAalifold [31], McCaskill-MEA [32] and Pfold [33].

To clarify the advantages and limitations of the present methods, their performances were assessed with the sum-of-pairs score (SPS), assuming that the manually curated alignment in Rfam [34] is correct. We also evaluated the accuracy of the secondary structures predicted from the resulting alignment. The accuracy of the secondary structure prediction can be affected by both the alignment process and the prediction process. The improvements in the prediction accuracy due to these two processes were separately assessed, by examining various combinations of alignment methods and structure prediction methods.

## Methods

### Algorithm

**Figure 1:**
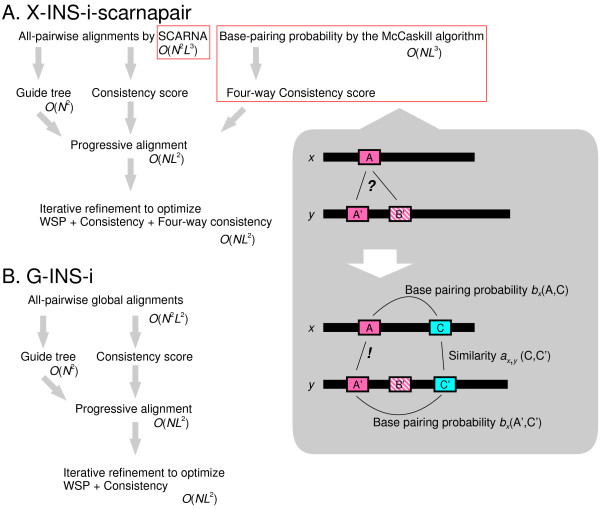
Schematic representation of the calculation procedure X-INS-i with Four-way Consistency (**A**) in comparison to that of G-INS-i (**B**).

The G-INS-i algorithm [22] is schematically illustrated in Fig. 1B. (*i*) All pairwise alignments are calculated by a global sequence alignment algorithm. (*ii*) Based on these alignments, an initial multiple alignment is built with the progressive method [35,36]. (*iii*) Then, the initial alignment is subjected to an iterative refinement process [20,37] to maximize an objective score. The objective function of G-INS-i has the Weighted sum-of-pairs (WSP [38]) term and the consistency [39] term that is derived from the pairwise alignments.

The proposed method, X-INS-i, is an extension with structural information incorporated into G-INS-i, as illustrated in Fig. 1A. The differences between X-INS-i and G-INS-i are the following two points: (*i*) X-INS-i uses a structural alignment method, instead of a global sequence alignment method, at the pairwise alignment stage. The present version uses either SCARNA [28] or LaRA [9]. (*ii*) In the progressive stage and the iterative refinement stage of X-INS-i, structural information is incorporated into the objective function through one more term, Four-way Consistency. The objective function for a group-to-group alignment between groups *X *and *Y *is represented as:

$$\text{Objective score}=\sum_{x\in X,y\in Y}w_{x}w_{y}(W_{m}{\text{ Pairwise score}}_{x,y}+W_{i}{\text{ Consistency}}_{x,y}+W_{r}{\text{ Four-way Consistency}}_{x,y}),$$

where *w*_*x *_is the weight for sequence *x *[36,38], and *W*_*m*_, *W*_*i *_and *W*_*r *_are weighting factors (*W*_*m *_= 2.0, *W*_*i *_= 3.2, *W*_*r *_= 8.0 in the current version). These three values were empirically determined mainly using 5S rRNA and tRNA sequences and structures [40,41], but have not finely tuned yet. With the second term, Consistency, a multiple alignment is evaluated with respect to its consistency with the pairwise alignments [39]. Unlike other consistency-based methods, such as TCoffee [27] and ProbCons [29], MAFFT (both G-INS-i and X-INS-i) uses only the primary library. The third term, Four-way Consistency, is derived from the base-pairing probability calculated from every single sequence as well as from every partly aligned group at each multiple alignment step.

The basic idea of Four-way Consistency is illustrated in the shaded area in Fig. 1. Let blocks A and A' be homologous to each other. When the evolutionary distance between the two sequences is large, A is sometimes misaligned because false homology, say B', occurred by chance. When blocks C and C', which are complementary to A and A', exist and are detected, the alignment between A and A' can be correctly recovered by using the information from the alignment between C and C'. In order to incorporate such information into an objective function, a combination of three types of the dynamic programming (DP) algorithm is performed at each node of the guide tree in both the progressive stage and the iterative refinement stage. We will first explain the algorithm for the pairwise alignment between sequences *x *and *y *and then clarify how to extend it to a group-to-group alignment. Let the lengths of sequences *x *and *y *be *l*_*x *_and *l*_*y*_, respectively.

1. For sequence *x*, the base-pairing probability *b*_*x*_(*i*, *j*) between residues *i *and *j *is calculated. Either the McCaskill algorithm [42], implemented in the Vienna RNA package [31], or the CONTRAfold algorithm [43], implemented in the CONTRAfold package [43], can be used for calculating *b*_*x*_(*i*, *j*).

2. The Vingron-Argos algorithm [44], which performs a global DP for both directions, is applied to sequences *x *and *y*. This algorithm produces an *l*_*x *_× *l*_*y *_matrix, in which the optimum alignment score $s_{x,y}^{\text{opt}}$ is assigned on the optimum path. On other elements (*i*, *k*) of the matrix, smaller values *s*_*x*, *y*_(*i*, *k*) are assigned as a result.

3. The similarity score *a*_*x*, *y*_(*i*, *k*) between residue *i *of sequence *x *and residue *k *of sequence *y *is defined as

$$a_{x,y}(i,k)=\max\left(\frac{s_{x,y}(i,k)}{s_{x,y}^{\text{opt}}},0\right).$$

4. Four-way Consistency, denoted as *Q*_*x*, *y*_(*i*, *k*), between residue *i *of sequence *x *and residue *k *of sequence *y *is defined as

$$Q_{x,y}(i,k)=\sum_{0<j<i,0<m<k}b_{x}(j,i)a_{x,y}(j,m)b_{y}(m,k)+\sum_{i<j\leq l_{x},k<m\leq l_{y}}b_{x}(i,j)a_{x,y}(j,m)b_{y}(k,m),$$

to which all possible base pairs contribute, according to their base-pairing probabilities.

5. A DP matrix is constructed using *Q*_*x*, *y*_(*i*, *k*) and two remaining terms to create an alignment between sequences *x *and *y*.

This procedure can be extended to a group-to-group alignment, as follows. The base-pairing probability *b*_*X*_(*i*, *j*) for group *X *is calculated as a weighted summation of *b*_*x *_of all sequences in group *X*:

$$b_{X}(I,J)=\sum_{x\in X}w_{x}b_{x}(i_{x}(I),j_{y}(J)),$$

where *w*_*x *_is the weight for sequence *x*, *i*_*x*_(*I*) is the residue number of aligned site *I *in sequence *x *and *j*_*y*_(*J*) is the residue number of aligned site *J *in sequence *y*. The similarity score *a*_*X*, *Y *_(*J*, *M*) between aligned site *I *of group *X *and aligned site *J *of group *Y *is obtained with a group-to-group alignment between groups *X *and *Y*. The Four-way Consistency score between the two groups is thus

$$Q_{X,Y}(I,K)=\sum_{0<J<I,0<M<K}b_{X}(J,I)a_{X,Y}(J,M)b_{Y}(M,K)+\sum_{I<J\leq l_{X},K<M\leq l_{Y}}b_{X}(I,J)a_{X,Y}(J,M)b_{Y}(K,M),$$

where *l*_*X *_and *l*_*Y *_are the alignment lengths of groups *X *and *Y*, respectively. At every step in the progressive and iterative refinement stages, a DP matrix is constructed using *Q *and the two remaining terms, and a group-to-group alignment is performed on it. The group-to-group alignment algorithm is the same as that for the sequence-based method used in other options of MAFFT [45].

### Implementation

The aforementioned algorithm was implemented as the X-INS-i option of the MAFFT sequence alignment program version 6.5. We used the McCaskill routine [42], taken from the Vienna RNA package [31] and McCaskill-MEA [32], to calculate the base-pairing probability. This routine can be replaced with an alternative method, CONTRAfold [43]. At the pairwise alignment stage, we used the SCARNA algorithm [28] implemented in the MXSCARNA [14] package for pairwise structural alignment. LaRA [9] can also be selected, instead of SCARNA. We chose SCARNA because it gave the best SPS score in the *N *= 2 subset of the BRAliBASE test. We also chose LaRA as an alternative pairwise alignment method, because it shows a characteristic pattern of the SPS score for pairwise alignment (*N *= 2) and it has a remarkably high structure conservation index [9,46,47]. Both MXSCARNA and LaRA can potentially compute a multiple alignment, but they are solely used as pairwise alignment methods.

The McCaskill algorithm consumes CPU time proportional to *L*^3^, where *L *is the sequence length. It runs *N *times for computing the Four-way Consistency, where *N *is the number of sequences. It also runs two times within the SCARNA routine, which is called *N*(*N *- 1)/2 times. In total, the McCaskill routine runs *N *+ *N*(*N *- 1) times. When SCARNA is used as a part of the proposed method, the McCaskill runs within the SCARNA part can potentially be omitted, because they are simply repetitions of the same calculation. If the repetitions are omitted, then the total number of McCaskill runs could be reduced to *N*. However, as the source of MXSCARNA is currently unavailable, we could not adopt this modification, and thus, used an *N *+ *N*(*N *- 1) version in this report. In the progressive alignment and iterative refinement stages, DP runs three times (in steps 2 and 5) at each node of the guide tree. Thus, these processes are approximately three times slower than the corresponding processes of G-INS-i, which has a time complexity of *O*(*L*^2^*N*^2^). Therefore, the overall time complexity of X-INS-i is *O*(*L*^3^*N*^2^) + *O*(*L*^2^*N*^2^) = *O*(*L*^3^*N*^2^), in the present implementation.

When LaRA is selected as the pairwise alignment algorithm, the present version of X-INS-i runs LaRA *N*(*N *- 1)/2 times. Thus, a part of LaRA calculation might be potentially omitted if the source of LaRA is open. In a preliminary experiment, however, it was not clear how much CPU time could be reduced by omitting the redundant calculation.

The experiments were performed on a Red Hat Enterprise Linux WS rel.4 on a 3.6 GHz Intel Xeon with 4 GB of RAM.

### Benchmark

There are presently about a dozen methods for global multiple ncRNA alignment. As representatives of the exiting methods, we selected the five latest methods, Murlet [10], MXSCARNA [14], RNA Sampler [11], LaRA [9] and MASTR [13], which reportedly outperformed the other methods, including FoldalignM [8], RNAcast [3], Dynalign [2] and Stemloc [4], consistently. As for RNA Sampler, both the default and fast options were examined. We also compared purely sequence-based methods, ClustalW [48], ProbConsRNA [29] and MAFFT-G-INS-i [22], in order to clarify the effect of the inclusion of structural information. In total, we tested these nine existing methods and the present method. The version number and the command-line arguments for each method are listed in Table 1.

**Table 1:** Version number and command-line arguments for each method

Method	Arguments
ClustalW 2.0 (iterative)	-Iteration = tree
ProbConsRNA 1.1	(default)
MAFFT-G-INS-i 6.516	mafft-ginsi
LaRA 1.3/1.31 *	(The default parameter file was used.)
Murlet 0.1	(default)
MXSCARNA 2	(default)
RNA Sampler 1.3	RNASampler_driver.pl -i 15 -S 100 > *output*
RNA Sampler 1.3 (fast)	RNASampler_driver.pl -i 15 -S 100 -f 1 > *output*
MASTR 1.0	(default)
X-INS-i-scarnapair 6.516	mafft-xinsi --scarnapair
X-INS-i-larapair 6.516	mafft-xinsi --larapair

* As the latest version of LaRA (1.31) frequently aborted for cases with more than two sequences, version 1.3 was used together with the parameter file of version 1.31.

We used two benchmark datasets, BRAliBASE version 2.1 [30] (referred to as BRAliBASE hereafter) and a dataset extracted from Rfam by Kiryu *et al*. [32] (referred to as the KKA dataset hereafter). For both datasets, the manually curated alignments in Rfam were assumed to be correct.

BRAliBASE was used for evaluating only the alignment accuracy, because it includes no reference structures. We used the compalignp program distributed at the BRAliBASE page [49] to calculate the sum-of-pairs score (SPS), which is defined as the fraction of pairs out of all possible character pairs that are aligned in both the predicted and reference alignments.

**Figure 2:**
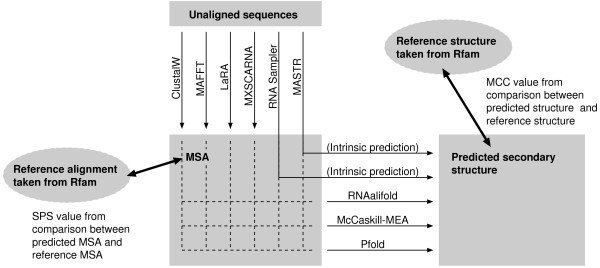
A flowchart of benchmarks using the KKA dataset.

The KKA dataset was used for assessing the accuracies of both alignment and consensus structure prediction. The KKA dataset is composed of the 17 RNA families listed in Table 2. A flowchart of benchmarks using the KKA dataset is shown in Figure 2. According to Kiryu *et al*. [32], each alignment was taken from Rfam seed alignments with the consensus structures published in the literatures. Note that we did not use sequence-based re-alignments, which were used by Kiryu *et al*. [32] in a different context. In addition to the alignment accuracy measured with the SPS score, the accuracy of secondary structure prediction was assessed as follows; after subjecting an alignment to three different prediction methods, Pfold [33], McCaskill-MEA [32] and RNAalifold [31], the predicted structures were compared to the Rfam structure. The internal predictions by RNA Sampler and MASTR were also compared. The difference between a predicted structure and the corresponding Rfam structure was evaluated with the MCC criterion [50],

**Table 2:** The KKA dataset.

Family name	Rfam accession #	Mean length	% identity
5S_rRNA	RF00001	116	57
5_8S_rRNA	RF00002	154	61
IRES_HCV	RF00061	261	94
Lysine	RF00168	181	49
RFN	RF00050	140	66
Retroviral_psi	RF00175	117	92
SECIS	RF00031	64	41
SRP_bact	RF00169	93	47
SRP_euk_arch	RF00017	291	40
S_box	RF00162	107	66
T-box	RF00230	244	45
THI	RF00059	105	55
U1	RF00003	157	59
U2	RF00004	182	62
UnaL2	RF00436	54	73
sno_14q_I_II	RF00181	75	64
tRNA	RF00005	73	45

Average		142	59

The length and identity values were taken from Table 1 in Kiryu *et al*. [32].

$$MCC=\frac{TP\times TN-(FP-\xi )\times FN}{\sqrt{\left(TP+FP-\xi )(TP+FN)(TN+FP-\xi )(TN+FN\right)}},$$

where *TP *is the number of 'true positives' (correctly predicted base-pairs), *FN *is the number of 'false negatives' (base-pairs in the reference structure that were not predicted), *TN *is the number of 'true negatives' (possible base-pairing interactions in a sequence that are not predicted and not in the reference structure, ie, pairs of nucleotide *xy *that are at least 4 nt apart, where *xy *∈ {AU, UA, CG, GC, UG, GU}.), *FP *is the number of 'false positives' (predicted base-pairs not in the reference structure) and *ξ *is the number of base-pairs that were incorrectly predicted but were *compatible *with the reference structure [15]. We used the compare_ct.pl program [51] for calculating *ξ*.

## Results

In comparison with G-INS-i, X-INS-i has two additional components, pairwise structural alignment and Four-way consistency, to incorporate the structural information, as illustrated in Fig. 1. In order to clarify the effect of each of the two components, we evaluated the accuracies of X-INS-i variants with and without the two components. We tested three different pairwise alignment methods (LaRA, SCARNA and a purely sequence-based method), and three different types of objective functions (with base-pairing probabilities by the McCAskill algorithm, with base-pairing probability by the CONTRAfold algorithm and with no base-pairing probability). There are thus nine possible variants in total.

The accuracy values of the nine variants are listed in Table 3. In both of the two criteria, SPS and MCC, the alignment accuracy was successfully improved by introducing Four-way Consistency, when comparing the accuracy values of variants with and without it. The difference between the McCaskill algorithm and CONTRAfold was unclear.

**Table 3:** Effects of two different parts that incorporate the structural information

			Accuracy of predicted structure (MCC)		
			
Structural pairwise alignment	Four-way consistency	SPS	Pfold	McCaskill-MEA	RNAalifold
Disabled (globalpair)	Disabled	0.768	0.622	0.646	0.622
Disabled (globalpair)	Enabled (McCaskill)	0.782	0.674	0.680	0.670
Disabled (globalpair)	Enabled (CONTRAfold)	0.781	0.665	0.675	0.668

Enabled (larapair)	Disabled	0.758	0.646	0.661	0.630
Enabled (larapair)	Enabled (McCaskill)	0.758	0.665	0.692	0.672
Enabled (larapair)	Enabled (CONTRAfold)	0.761	0.661	0.689	0.677

Enabled (scarnapair)	Disabled	0.787	0.699	0.687	0.693
Enabled (scarnapair)	Enabled (McCaskill)	0.789	0.724	0.712	0.726
Enabled (scarnapair)	Enabled (CONTRAfold)	0.794	0.711	0.705	0.704

The KKA dataset was used as the benchmark. The accuracies of alignments measured by the SPS criterion are listed in the SPS column. The accuracies of predicted common secondary structures are shown in the three columns on the right. The alignment by each method was subjected to three external prediction programs, Pfold, McCaskill-MEA and RNAalifold, and then the differences from the Rfam curated structure were calculated with the MCC criterion.

Among the three different types of pairwise alignment routines (SCARNA, LaRA and the sequence-based method), SCARNA was better than LaRA and the sequence-based method. The difference between SCARNA and LaRA probably reflects the difference in the the accuracy of pairwise alignment: according to the *N *= 2 subset of BRAliBASE, the average SPS score of SCARNA is higher than that of LaRA.

Based on this result, we decided to use mainly the X-INS-i algorithm with the combination of SCARNA and McCaskill, which is referred to as X-INS-i-scarnapair hereafter. As SCARNA internally uses the McCaskill algorithm, this combination keeps the method internally consistent. Moreover, the McCaskill algorithm is somewhat faster than CONTRAfold with similar accuracy. We also examined the combination of LaRA and McCaskill, which is referred to as X-INS-i-larapair, in order to compare the direct application of LaRA for multiple alignment problems and the use of the LaRA pairwise alignment within the X-INS-i framework.

**Figure 3:**
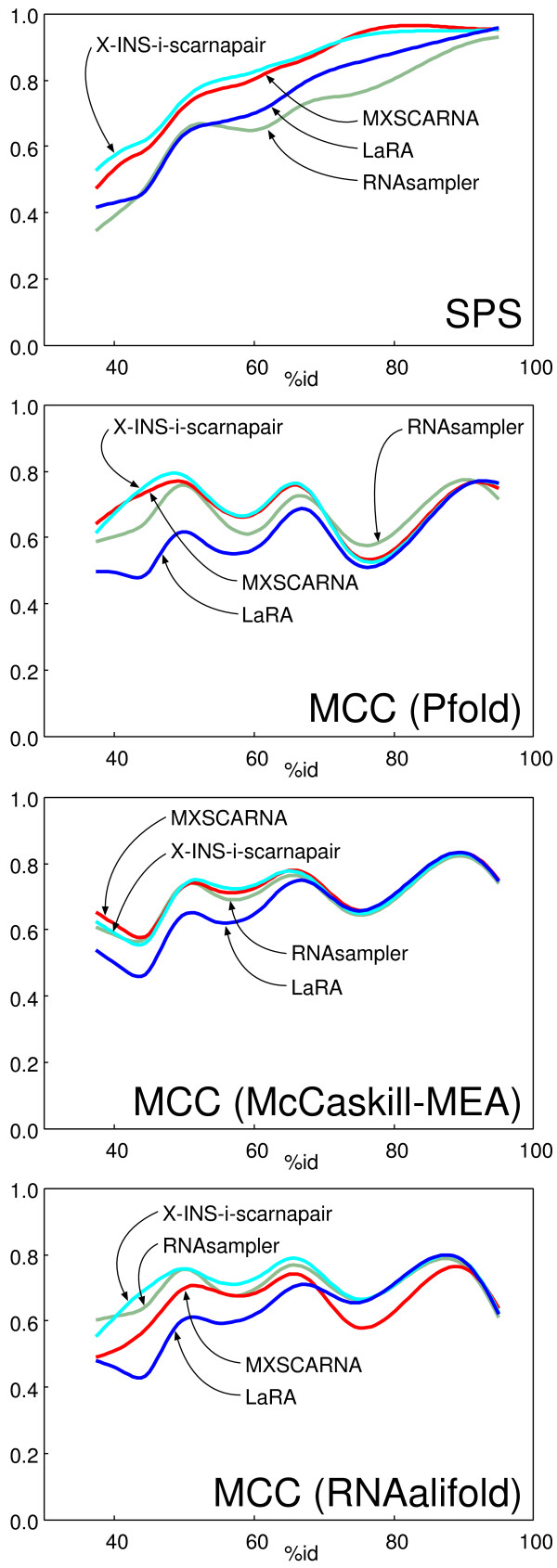
Accuracy of alignment and structure prediction as a function of the average percent identity among input sequences. The KKA dataset was used. The alignment and structure in Rfam were assumed to be correct and the difference from them were estimated with the SPS (for assessing the alignment accuracy) and MCC (for assessing the accuracy of structure prediction). The programs used for predicting secondary structure are indicated in parentheses. The percent identities were calculated from the reference alignments. The curves were fitted using a cubic spline.

Table 4 shows the results of the KKA test. The two types of accuracy values, SPS and MCC, of X-INS-i-scarnapair and X-INS-i-larapair were compared with those of the existing methods. X-INS-i-scarnapair was the best in the SPS criterion. When the alignments were subjected to three different secondary structure prediction programs, X-INS-i-scarnapair was the best but other methods, RNA Samper and MXSCARNA, also performed well when McCaskill-MEA is used for predicting the structure. Fig. 3 shows two types of accuracy values, SPS and MCC, as a function of similarity among input sequences. The SPS score of X-INS-i-scarnapair was consistently higher than those of the other methods, while the MCC scores of X-INS-i-scarnapair were comparable to or slightly higher than other accurate methods, RNA Sampler and MXSCARNA. As references, the accuracies of the internal predictions by RNA Sampler and MASTR are shown in the 'intrinsic' column. In this test, the advantage of intrinsic prediction was unclear.

**Table 4:** Comparison to existing methods

			Accuracy of predicted structure (MCC)			
			
Method	Time (s.)	SPS	Pfold	McCaskill-MEA	RNAalifold	(intrinsic)
ClustalW (iterative)	98	0.669	0.488	0.554	0.482	
ProbConsRNA	61	0.763	0.654	0.651	0.613	
G-INS-i	12	0.768	0.622	0.646	0.622	

LaRA 1.31	15,000	0.687	0.607	0.649	0.600	
Murlet	64,000	0.773	0.712	0.702	0.668	
MXSCARNA 2	700	0.769	0.718	**0.712**	0.666	
RNA Sampler (fast)	19,000	0.641	0.659	0.684	0.662	0.655
RNA Sampler	70,000	0.655	0.685	**0.703**	0.705	0.705
MASTR	24,000	0.662	0.570	0.616	0.592	0.601
X-INS-i-larapair	15,000	0.758	0.665	0.692	0.672	
X-INS-i-scarnapair	1,800	**0.789**	**0.724**	**0.712**	**0.726**	

The KKA dataset was used as the benchmark. The accuracies of alignments measured by the SPS criterion are listed in the SPS column. The SPS value was computed for each alignment and then averaged across all the alignments. The accuracies of predicted common secondary structures are shown in the four columns on the right. The alignment by each method was subjected to three external prediction programs, Pfold, McCaskill-MEA and RNAalifold, and then the differences from the Rfam curated structure were assessed. The MCC values were computed for each sequence and then averaged across all the sequences. The accuracy values for secondary structure internally predicted by RNA Sampler and MASTR are shown in the (intrinsic) column. The highest score in each column is underlined. The scores close to the highest (*p *> 0.01 in the Wilcoxon test) are shown in bold. McCaskill-MEA was run with the default value *α *= 0.91.

The MCC values of MASTR was not so high in the KKA test. This contradicts Lindgreen *et al*. [13], in which the accuracy of MASTR is comparable to or only slightly lower than that of RNA Sampler. We also performed a benchmark test using the dataset collected by them and confirmed that their result was reproduced and that X-INS-i-scarnapair outperformed both RNA Sampler and MASTR (see supplemental data [52]). Probably, an alignment problem that is easily solved by a method is not always an easy problem for another method. It may be meaningful to provide a guideline to select an appropriate method, by testing which method is suitable for which type of problem.

**Figure 4:**
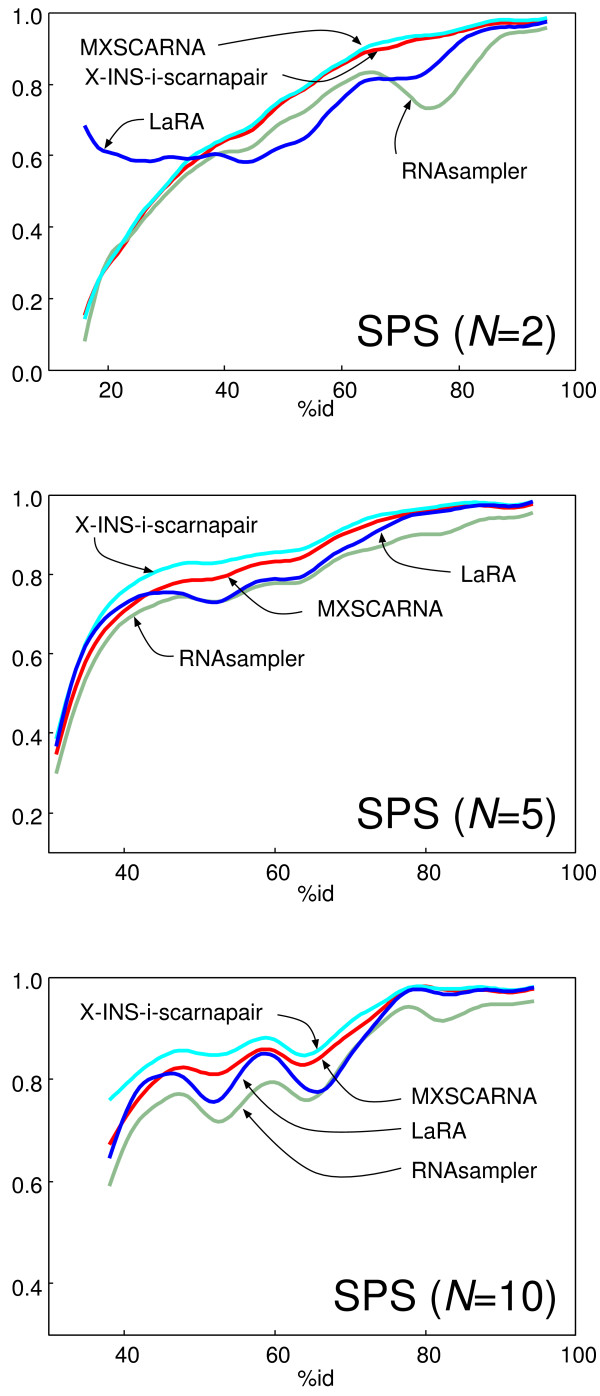
SPS values as a function of the precent identity among input sequences. The BRAliBASE dataset was used. The percent identities given with the dataset were used. The curves were fitted using a cubic spline.

Table 5 shows the results of the BRAliBASE benchmark. When the number of sequences is two (*N *= 2), the accuracy of X-INS-i-scarnapair was close to that of MXSCARNA. This is expected, because these two methods employ the same pairwise alignment by SCARNA. In all of the other cases (*N*>2), X-INS-i-scarnapair significantly outperformed MXSCARNA. A similar result was obtained when comparing LaRA and X-INS-larapair; the latter outperformed the former. The SPS score of X-INS-i-scarnapair was higher than that of X-INS-i-larapair, with statistical significance in many cases. The difference probably reflects the difference between SCARNA and LaRA in the pairwise alignment stage. Fig. 4 shows the SPS values as a function of similarity among the input sequences. In the case of pairwise alignment (*N *= 2), the SPS score of LaRA was remarkably high for diverged sequences with a percent identity of ~40%, whereas the SPS score of X-INS-i-scarnapair was the highest for other cases.

**Table 5:** SPS scores for BRAliBASE version 2.1

		SPS					
		
Method	Time	*N *= 2	*N *= 3	*N *= 5	*N *= 7	*N *= 10	*N *= 15
ClustalW (iterative)	52 minutes	0.796	0.810	0.828	0.837	0.850	0.853
ProbConsRNA	33 minutes	0.836	0.855	0.879	0.890	0.899	0.907
G-INS-i	8.8 minutes	0.837	0.851	0.874	0.890	0.901	0.913

LaRA 1.31	5.5 days	0.798	0.830	0.864	0.883	0.898	0.913
Murlet	2.5 weeks	0.843	0.863	0.886	0.897	0.906	0.915
MXSCARNA 2	4.2 hours	0.850	0.866	0.884	0.894	0.907	0.914
RNA Sampler (fast)	8.2 days	0.787	0.801	0.824	0.828	0.841	0.855
RNAsampler	2.9 weeks	0.785	0.812	0.839	0.850	0.858	0.869
X-INS-i-larapair	5.4 days	0.837	0.869	0.896	0.909	0.919	**0.930**
X-INS-i-scarnapair	18 hours	**0.856**	**0.876**	**0.902**	**0.913**	**0.922**	**0.931**

# of alignments		8,976	4,835	2,405	1,426	845	503
(used for Wilcoxon test)		(8,976)	(4,832)	(2,399)	(1,420)	(836)	(491)

The highest scores within each group (*N *= 2, 3, 5, 7, 10, 15) are underlined. The scores close to the highest (*p *> 0.01 in the Wilcoxon test) are shown in bold. As Murlet and RNA Sampler aborted for a small number of datasets, the Wilcoxon test was carried out using a limited set (the numbers of alignments are in parentheses), for which every method returned an alignment.

## Discussion

Since MXSCARNA and X-INS-i-scarnapair use the same pairwise alignment algorithm, SCARNA, the difference in the accuracy between them should reflect the difference in the multiple alignment part. In all of the tests with more than two sequences, X-INS-i-scarnapair significantly outperformed MXSCARNA in the SPS criterion. The difference should be interpreted as the improvement achieved by the present framework. The same observation was made with the comparison between LaRA and X-INS-i-larapair. The improvements in the accuracy from LaRA to X-INS-i-larapair should reflect those introduced by the present method.

MXSCARNA seems to use the ProbCons framework to extend the SCARNA pairwise alignments to a multiple alignment. LaRA combines pairwise structural alignments into a multiple alignment using TCoffee. In contrast, X-INS-i uses the base-pairing probability of every aligned group at every step of the progressive and iterative refinement stages. RNA Sampler also incorporates the base-pairing probability into the iterative refinement stage. Our experiments suggest that the simple combination of a pairwise structural alignment algorithm and existing multiple sequence alignment framework is insufficient and that it is important to incorporate the structural information at the multiple alignment stage.

X-INS-i uses structural information at two components: (*i*) the pairwise alignments and (*ii*) the Four-way Consistency objective function. The improvement solely by the latter can be assessed with the second and third raws in Table 3, in which only the Four-way Consistency objective function was used but no pairwise structural alignment was performed. Interestingly, by incorporating the base-pairing probability for every sequence into the iterative refinement step through the new objective function, a considerable improvement in the accuracy was observed. We made this method selectable as the Q-INS-i option.

The present methods, both X-INS-i and Q-INS-i, output a multiple sequence alignment but predicted structural information is hidden in the output. The process to infer a structure from the base-pairing probability is left to external programs. Within the present method, the structural information is always used in the form of base-pairing probability, but not as a single structure. This strategy has merit, since we can keep the ambiguity of the prediction during the alignment process, whereas the present methods cannot calculate an alignment assuming a single optimum secondary structure deterministically predicted. At this point, it is unclear which type of multiple alignment strategy is better: to align sequences assuming a single secondary structure or to align sequences based on an ambiguous secondary structure. We designed the present method based on the presumption that the secondary structure may have undergone small changes in the course of evolution, and so it may be difficult to determine a single structure for a set of diverged ncRNA sequences. Accordingly, in equation 1, all possible base pairs are summed to contribute to the objective function along with the base-paring probabilities.

When we evaluated the accuracy of a common secondary structure, we examined the effects of the alignment method and the structure prediction method separately. The results suggest that, at present, the quality of the alignment affects the prediction accuracy much more than the selection of a structure prediction program does, and that external prediction programs perform slightly better than prediction functions internally implemented in the currently available RNA alignment methods. These observations are consistent with those of previous studies [10,32].

Pseudoknots were not considered at all in the present analyses. If a method to compute the base-pairing probability also considers pseudoknots, then it can be incorporated into the present formulation.

## Conclusion

X-INS-i builds a multiple structural RNA alignment incorporating secondary structural information of aligned groups at every step of the progressive and iterative refinement processes, through the Four-way Consistency objective function. In the SPS criterion, X-INS-i-scarnapair, a combination of X-INS-i with SCARNA, significantly outperformed existing methods. As a basis of common secondary structure prediction, the quality of the X-INS-i-scarnapair alignment was estimated to be comparable to or somewhat higher than those of existing methods.

The current version of X-INS-i-scarnapair is faster than RNA Sampler and Murlet, but slower than MXSCARNA. If the source of MXSCARNA becomes open, then the time complexity of X-INS-i-scarnapair can be reduced from *O*(*L*^3^*N*^2^) to *O*(*L*^3^*N*) + *O*(*L*^2^*N*^2^), as explained in the Implementation section.

Any type of pairwise structural alignment can be incorporated into the present method simply by adjusting the input/output format, to build a multiple alignment. The latest version of FOLDALIGN (version 2.1.0, published 2007 Oct. [53]), which is based on the Sankoff algorithm, can be selected instead of SCARNA and LaRA. Both the local and global options of FOLDALIGN are selectable with the --foldalignlocalpair and --foldalignglobalpair options, respectively. If a new method for computing base-pairing probability is developed in the future, then it can also be incorporated into the present framework.

## Supplementary data

Additional benchmark results are available at [52].

## Availability and requirements

Project name: MAFFT

Project home page: 

Operating systems: Mac OS X, Windows (requires Cygwin), Linux, UNIX

Programming languages: C, C++

License: The code for the McCaskill algorithm was taken from RNAalifold [31] and McCaskill-MEA [32]. This part should be distributed under their own licenses. The remaining part is under the BSD license.

## Authors' contributions

KK conceived the study and developed the computer program. KK and HT wrote the manuscript. Both authors read and approved the final manuscript.
